# Establishment of Regeneration Propagation Protocol Based on In Vitro Seeds Development of the Endangered Orchid Species *Dendrobium flexicaule*

**DOI:** 10.3390/plants15152318

**Published:** 2026-07-28

**Authors:** Guoye Guo, Yichen Liu, Manyu Su, Xiaoyi Jing, Pengfei Li, Yicai Zhan, Yuhan Jiang, Yuxi Wu, Xinyuan Hou, Bo Li, Xiaomin Hu, Lunguang Yao

**Affiliations:** 1 College of Water Resources and Modern Agriculture, Nanyang Normal University, Nanyang 473061, China; 2 College of Life Sciences, Henan University, Kaifeng 475004, China; 3 Henan Field Observation and Research Station of Headwork Wetland Ecosystem of The Central Route of South-to-North Water Diversion Project, Nanyang Normal University, Nanyang 473061, China

**Keywords:** *Dendrobium flexicaule*, tissue culture, seed germination, protocorm, in vitro rooting

## Abstract

*Dendrobium flexicaule* has extremely strict requirements for its habitat, and its seeds are small and lack stored nutritional reserves, resulting in an extremely low natural germination rate. Excessive pursuit and excavation of wild *D. flexicaule* have led to the gradual depletion and a critical situation of endangerment. This study conducted tissue culture and in vitro regeneration of mature seeds of the endangered medicinal plant *D. flexicaule*, systematically investigating the optimal culture conditions for the regeneration propagation system. The results showed that, for the primary culture of sterile germination in the seed stage, the optimal culture medium was MS medium containing 0.2 mg/L NAA and 80 g/L potato homogenate, with the highest germination rate of 98.33%, and the protocorms grew robustly. The optimal subculture for the proliferation and differentiation of protocorms was MS medium containing 0.5 mg/L NAA and 0.5 mg/L 6-BA, supplemented with 0.5 g/L activated carbon, with a proliferation coefficient of 1.88 and differentiation rate of 81.33%; the clustered buds developed well. The 1/2 MS medium with added 50 g/L banana puree and 1 g/L activated carbon significantly promoted the growth of tissue culture seedlings, suggesting that medium without the addition of plant growth regulators is the optimal formulation for promoting vigorous seedling growth and rooting induction of *D. flexicaule* tissue culture seedlings. This study established an effective protocol for in vitro tissue culture of *D. flexicaule*, which is expected to offer valuable technical support for the establishment of rapid propagation and cultivation, relieving its endangered situation of wild resources.

## 1. Introduction

*Dendrobium flexicaule* Z. H. Tsi, S. C. Sun & L. G. Xu, a perennial herb of the genus *Dendrobium* in the family Orchidaceae Juss. [1,2,3,4,5]. The wild populations of *D. flexicaule* are mainly distributed in Henan, Hubei, Sichuan, Chongqing, and Guizhou, China, and are commonly found on damp rocks at altitudes ranging from 1200 to 2000 m [5,6,7,8]. *D. flexicaule* has unique ecological habits, preferring warm and humid conditions, and its wild resources are distributed in a narrow range [7,8,9]. *Dendrobium* has significant medicinal value and a long history of application in China. In traditional Chinese medicine, the fresh or dried stems of *Dendrobium* are generally used as medicine, which are rich in various functional active substances such as polysaccharides, amino acids, and alkaloids, and are an important source of the traditional precious Chinese medicine, Xi Fengdou [5,7,8,10,11]. About 2300 years ago, the earliest existing Chinese pharmacological monograph, “*Shen Nong’s Materia Medica*”, recorded that *Dendrobium* was sweet in taste and had the functions of nourishing the stomach, promoting the secretion of body fluids, nourishing yin, clearing heat, and improving eyesight [8,10,12,13]. The monumental pharmacological work “*Compendium of Materia Medica*” of the Ming Dynasty in China recorded that *Dendrobium* had the effects of strengthening the kidneys, benefiting essence, thickening the intestines and stomach, regulating the stomach qi, enhancing intelligence, dispelling fright, and lightening the body and prolonging life [12,13]. The “*Compendium of Chinese Medicinal Materials Resources—Chongqing Volume*” published in 2021 listed *D. flexicaule* as a rare and endangered medicinal material, noting its efficacy in benefiting the stomach, promoting the production of body fluids, nourishing yin and clearing heat. In 2025, the “*Pharmacopoeia of the People’s Republic of China*” issued by the National Medical Products Administration and the National Health Commission of China once again included *Dendrobium* as a Chinese medicinal material (https://www.gov.cn/zhengce/zhengceku/202503/content_7015682.htm, accessed on 20 September 2025). In folk medicine, *D. flexicaule* has long been regarded as a more valuable Chinese medicinal material than common *Dendrobium* varieties such as *Dendrobium officinale*, and the market price of its wild fresh products is much higher than that of other *Dendrobium* species or artificially cultivated ones. The hype in the Chinese medicinal materials market has driven excessive pursuit and excavation of wild *D. flexicaule*, leading to the gradual depletion of its wild resources and a critical situation of endangerment [10]. In 2021, the National Forestry and Grassland Administration of China listed *D. flexicaule* as a first-class protected wild plant and included it in the “*List of Key Protected Wild Plants of the People’s Republic of China*” (https://www.forestry.gov.cn/c/www/lczc/10746.jhtml, accessed on 20 September 2025), and it was also listed as an endangered plant by the International Union for Conservation of Nature (IUCN) (https://www.iucnredlist.org, accessed on 20 September 2025).

*D. flexicaule* is a precious medicinal plant and ornamental flower plant with significant market potential and development value [8,13]. Currently, the research on *D. flexicaule* mainly focuses on its systematic evolutionary studies [1,2,3,4,5,13,14,15] and pharmacologically active substances [16,17,18,19,20]. Due to the limited wild resources of *D. flexicaule* and its status as a national first-class protected plant in China, it is difficult to meet market demand. As an epiphytic orchid plant, *D. flexicaule* has extremely strict requirements for its habitat, and its seeds are small and lack stored nutritional reserves, resulting in an extremely low natural germination rate [21,22]. Artificial cultivation and propagation of *D. flexicaule* is the key to solving the endangered situation of wild resources and the insufficient supply of medicinal materials in the market. Artificial cultivation of *D. flexicaule* mainly relies on division propagation, but the breeding capacity is low, and the growth is slow [23]. Compared with traditional breeding techniques, plant tissue culture technology has a short cycle, fast breeding speed, high proliferation rate, controllable culture conditions, and is widely used in the research fields of rare flower propagation, plant germplasm resource protection, new variety breeding, and genetic improvement [24,25,26,27,28,29]. Plant tissue culture technology also provides important technical support for the conservation and sustainable utilization of orchid genus *Dendrobium* plants with significant ornamental and medicinal values. Research on rapid in vitro propagation of the genus *Dendrobium* plants has made certain progress [30,31,32,33], but there is a lack of systematic research on tissue culture of *D. flexicaule*, and the culture medium formulas among different studies vary greatly [21,22], which is not conducive to the production application of *D. flexicaule* and the large demand for Chinese medicinal herb “Feng Dou” of *D. flexicaule* in the Chinese medicinal materials market.

The sterile tissue culture using seeds as explants and the cultivation of regenerated seedlings are effective ways to achieve rapid plant propagation [34,35,36,37]. Under sterile conditions, by providing nutrients and plant hormones to the orchid seeds through an MS medium supplemented with nitrogen sources, carbon sources, and plant growth regulators (PGR), and relying on controllable environmental conditions (temperature, light, humidity), the non-symbiotic germination of orchid species seeds of the genus *Dendrobium* can be promoted [38,39,40,41]. Currently, there are relatively few research reports on endangered medicinal plants such as *D. flexicaule*. This study focuses on the sterile germination of *D. flexicaule* seeds, the proliferation and differentiation of protocorms, and the growth and rooting of regenerated seedlings in three key cultivation stages. Using MS as the basic medium, by designing different types and contents of exogenous plant hormones and different contents of exogenous additives, and optimizing and screening the best medium formula, an in vitro tissue culture and rapid propagation system based on *D. flexicaule* seeds could be established. This is conducive to protecting the wild germplasm resources of *D. flexicaule* and the diversity of the genus *Dendrobium*, alleviating the crisis of the endangered wild *D. flexicaule* resources, and promoting the sustainable utilization and development of medicinal resources.

## 2. Results

### 2.1. Seed Germination and Protocorms Induction

**Figure 1:**
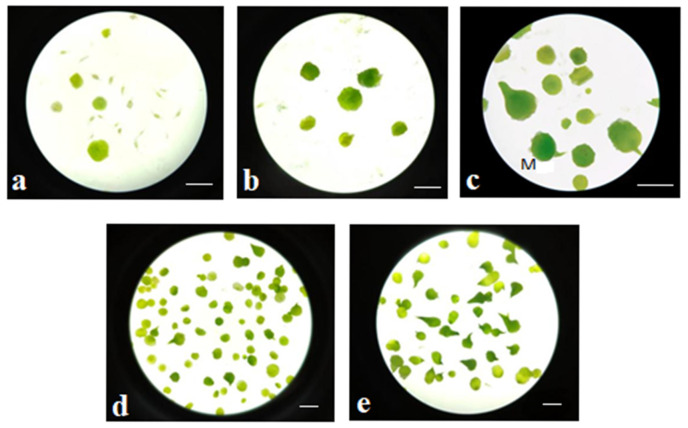
The sterile germination process of the seed of *Dendrobium* flexicaule. (**a**) The long spindle-shaped seeds of the embryo before germination; (**b**) differentiate into protocorms; (**c**) M represents protocorms with “absorptive epidermal hairs” present; (**d**) pointed conical part at the top of the protocorm is the leaf primordium formed by differentiation; (**e**) protocorms differentiated into young shoots. Scale bar (**a**–**b**) = 0.5 mm, scale bar (**c**–**e**) = 1.0 mm.

After inoculating the seeds of *D. flexicaule* onto the explant induction medium for two weeks, no contamination was found in any of the culture media; the contamination rate was 0, indicating excellent disinfection efficacy. Regular microscopic observations of the micro-morphology were conducted using an optical microscope. It was found that the germination status of the seeds and the growth of the explants varied under different medium formulations. The mature seeds of *D. flexicaule* are spindle-shaped and have a seed embryo, which is covered by a layer of transparent pericarp. Through the observation of the experimental materials during cultivation, it was discovered that the morphological characteristics of the seeds of *D. flexicaule* during germination are as follows: Firstly, they need to absorb sufficient water; the seeds gradually change from light yellow–green to light green; then the seed embryo breaks through the pericarp and the seeds start to germinate; subsequently, an explant forms, and the pericarp gradually disappears. The mature explants of *D. flexicaule* have obvious polarity at both ends, and the protruding part at the top of the explant continuously elongates and eventually differentiates into young leaves (Figure 1). After approximately two months of in vitro induction culture, the growth and development of *D. flexicaule* seeds under different culture medium formulations were observed and statistically analyzed (Table 1), including the germination time, germination rate, and growth morphology of protocorms. It was observed that the seeds of *D. flexicaule* can germinate under different culture media, but the degree of germination varies. Among them, the seeds in No. 2 culture medium showed the most obvious germination, with rapid germination, dark green color, and vigorous growth.

By investigating the effects of different concentrations of NAA and exogenous additives on the germination of *D. flexicaule* seeds, the results (Table 1) showed that adding 0.2 mg/L NAA and 80 g/L potato homogenate to the MS medium results in the best seed germination effect, with the highest seed germination rate of 98.33%, the fastest germination speed, and the differentiated protocorms with vigorous growth are plump and dense. After 15 days of sterile culture, the seeds began to germinate successively and entered the morphological formation stage. Initially, a conical protocorm structure was formed. In the blank control group (CK, without adding NAA and potato homogenate), most of the embryos showed abnormal differentiation, presenting as tissue callus formation at the base and difficulty in forming a typical protocorm structure. By comparing the induction results of culture media 1 and media 3 with those of culture media 2 and media 4, it was known that when the amount of potato homogenate added was constant, compared with 0.5 mg/L NAA, adding 0.2 mg/L NAA resulted in a significantly shorter germination time of the *D. flexicaule* seeds in the culture medium, with the protocorm turning green and growing faster. This difference verified that exogenous organic additives and plant hormones have a positive regulatory effect on the germination of *D. flexicaule*.

### 2.2. Protocorms Proliferation and Differentiation

**Figure 2:**
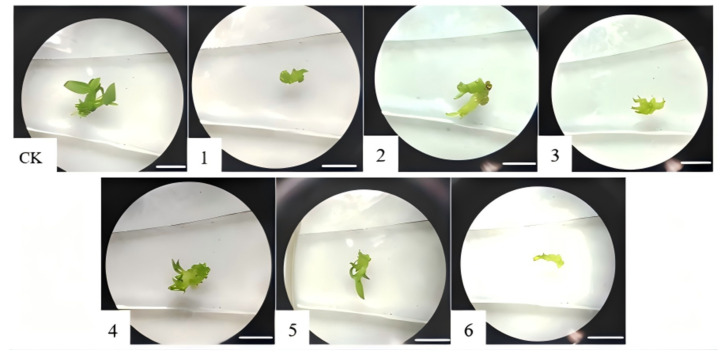
Proliferation and differentiation of protocorms of *D. flexicaule* in different culture medium treatments after 45 days of cultivation (10×). The number in the lower left corner represents the number of different culture medium treatments. Scale bar = 1.0 cm.

**Figure 3:**
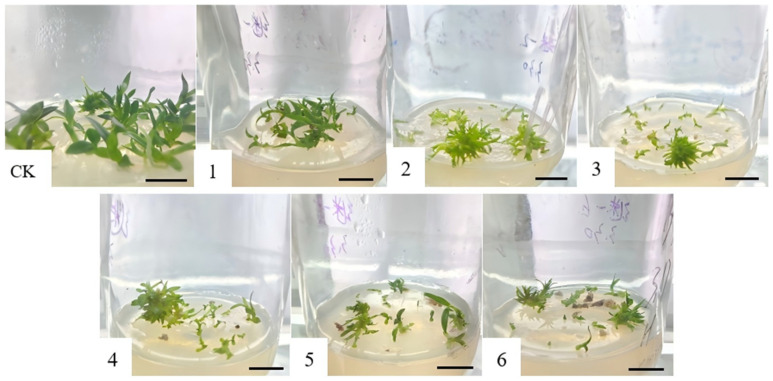
Effects of different concentrations of 6-BA combined with NAA on the proliferation and differentiation of protocorms after two months of cultivation. The number in the lower left corner represents the number of different culture medium treatments. Scale bar = 1.0 cm.

The protocorms of *D. flexicaule* were transplanted onto the proliferation induction medium, and after 45 days of cultivation, it was observed that the clustered buds proliferated more significantly in the 2nd and 4th media (Figure 2). The clustered buds in the 4th medium proliferated the most, with normal color and fullness, while the clustered buds proliferated less in the other media. After 2 months of protocorm cultivation, all 7 treatments resulted in varying degrees of proliferation and differentiation of protocorms. The number, length, and growth vigor of the clustered buds of *D. flexicaule* in each medium increased significantly (Figure 3 and Table 2). The data showed that when NAA is 0.2 mg/L, as the concentration of 6-BA increases, the proliferation coefficient of the protocorm first increases and then decreases; when NAA is 0.5 mg/L, as the concentration of 6-BA increases, the proliferation coefficient of protocorms first decreases and then increases. When the concentration of 6-BA is 0.5 mg/L, the proliferation coefficient of protocorms is the highest at 1.88, and the clustered buds are dark green, plump, and have the best growth condition. When the concentration of 6-BA is 0.5 mg/L and 1.5 mg/L, respectively, higher NAA concentrations resulted in greater multiplication coefficients of protocorms; but when the concentration of 6-BA is 1.0 mg/L, the proliferation coefficient of protocorms decreases with the increase in the NAA concentration. Among them, the proliferation coefficient of protocorms in the CK group without any addition of plant growth regulators is the lowest at 0.77, indicating that adding an appropriate amount of plant hormones can promote the proliferation of protocorms of *D. flexicaule*. This research also found that the effect of protocorm differentiation into shoots of *D. flexicaule* varied significantly among different medium treatments as the concentration ratios of NAA and 6-BA changed (Figure 3 and Table 2). In the CK control treatment without exogenous plant hormones, the protocorm differentiation rate reached 100%, with all inoculated protocorms differentiating into seedlings that were dark green and robust, and the differentiated seedlings grew vigorously and produced abundant adventitious roots (Figure 3—CK).

Based on the observational data, when the NAA concentration was constant, the differentiation rate of protocorms gradually decreased with the increase in the 6-BA concentration. When the 6-BA concentration was constant, a low concentration of NAA (0.2 mg/L) was more conducive to promoting bud differentiation of protocorms of *D. flexicaule*. Among them, most of the protocorms inoculated with 1st and 4th culture medium were able to differentiate into buds, with differentiation rates reaching 86% and 82%, respectively. The buds were robust, and the leaf color was emerald green. Comprehensive comparative analysis showed that there were significant differences in the protocorm proliferation coefficients among culture media 2, media 4, media 1, and media CK (control group). The proliferation and differentiation effect of protocorms is the best when the MS medium is supplemented with 0.5 mg/L 6-BA and 0.5 mg/L NAA, with the proliferation coefficient being the highest at 1.88 and the differentiation rate reaching 81.33%, and the protocorms have dense proliferation, dark green color, plump individuals, and mostly differentiate into bud seedlings, with the seedlings being strong. The two-way ANOVA of variance further substantiated that (Table 2), the interaction effect of NAA and BA on the proliferation coefficient was significant (F = 5.917, *p* < 0.05), indicating that the combined concentrations of the two hormones have a synergistic regulatory effect on the proliferation of the protocorms. Different concentrations of NAA and 6-BA have a significant effect on the differentiation of the protocorms.

### 2.3. Vigorous Growth and In Vitro Rooting

**Figure 4:**
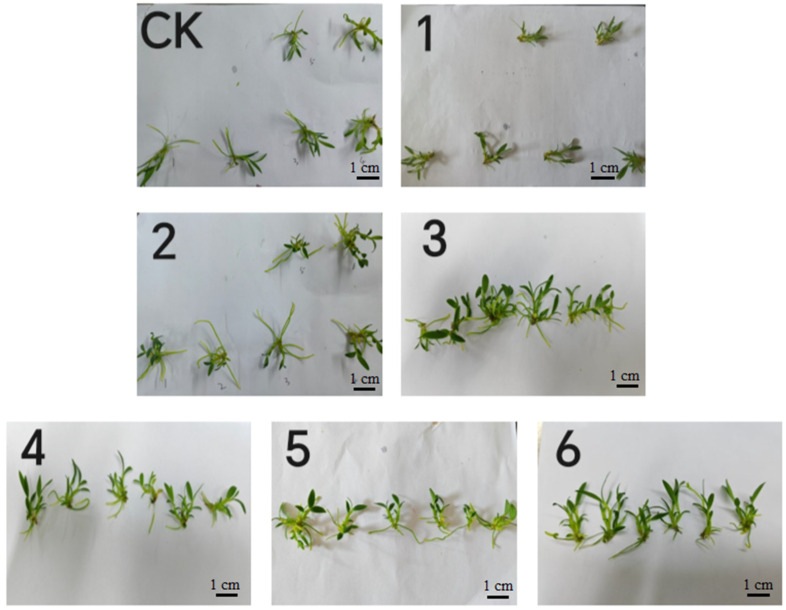
Effects of different concentrations of plant growth regulators on vigorous growth and Rooting. The number in the top left corner represents the number of different culture medium treatments. Scale bar = 1.0 cm.

After a 60-day cultivation cycle, the growth conditions of tissue culture seedlings of *D. flexicaule* showed significant changes in the number of roots and rooting percentage (%), root length, number of adventitious buds, number of leaves and plant height, and overall plant growth (Figure 4, Table 3). At the end of the second week of cultivation, some materials at the base began to develop conical differentiation protrusions; continuing the cultivation to the third week, these protrusions developed into white adventitious roots, with a length of 1 to 3 cm, and the surface was densely covered with root hairs. After 21 days of cultivation, it was observed that the tissue culture seedlings in the CK medium, No. 3 medium, and No. 5 medium had relatively better growth than seedlings in other media, with fast root proliferation, vigorous plant growth, and many leaves; while the tissue culture seedlings in the other treatment media had slow root development and insufficient biomass accumulation, indicating that the composition of the medium has an important regulatory effect on the morphological formation of *D. flexicaule* tissue culture seedlings.

Analysis of one-way ANOVA indicated (Table 3) that there were significant differences in the effects of different combinations and concentrations of plant growth regulators on the growth and root induction of tissue culture seedlings of *D. flexicaule*. The differences were manifested in the average number of roots per cluster of shoots, root length, the number of proliferating buds, and the number of leaves per explant. By comparing the induction conditions of culture media 1, media 2, and media 3, it was found that when the concentration of 6-BA was 0.5 mg/L, with the increase in the NAA concentration, the average number of roots per cluster of shoots, root length, and number of leaves per cluster all showed a significant increasing trend. By comparing the induction conditions of culture media 4, media 5, and media 6, when the concentration of 6-BA was 1 mg/L, with the increase in the NAA concentration, the average number of roots per shoot, average root length, number of proliferating buds, and average leaf number also increased, but the plant height did not change significantly. The two-way ANOVA of variance further substantiated that (Table 3), the interaction effects of NAA and BA were extremely significant for the average number of roots (*p* = 0.000), root length (*p* = 0.000), and number of leaves per cluster of shoots (*p* = 0.001), indicating that the response to increasing NAA and 6-BA concentration differed in supplemented media.

Comprehensive analysis showed that when the concentration of 6-BA increased from 0.5 mg/L to 1 mg/L, the average number of roots and average root length of the shoot clusters showed a downward trend, indicating that NAA can effectively promote the rooting of tissue culture seedlings of *D. flexicaule*, while 6-BA significantly antagonized adventitious rooting. The results showed that medium CK has a more prominent effect in promoting the growth of tissue culture seedlings. The control treatment achieved 100% rooting, with 5.09 ± 0.38 roots per shoot which only slightly lower than media 5, the prominent average root length of 2.03 ± 0.15 cm and average adventitious buds of 1.70 ± 0.77, as well as modest number of leaves per shoot (12.24 ± 0.37) and plant height (2.37 ± 0.39 cm), which demonstrates an inherent capacity for in vitro rooting. In addition, when the medium was supplemented with 1.0 mg/L 6-BA and 0.2 mg/L NAA, the rooting rate of *D. flexicaule* was 100%, the average number of roots per shoot was the largest, and the root length, number of leaves, and plant height showed moderate performance. Based on the results of the vigorous growth and In Vitro rooting experiments and the above analysis, 1/2 MS as the basic medium, adding banana puree and PGR-free, significantly promoted the growth of tissue culture seedlings, suggesting that medium without the addition of plant growth regulators is the optimal formulation for promoting vigorous seedling growth and rooting induction of *D. flexicaule* tissue culture seedlings.

## 3. Discussion

*D. flexicaule* has strict habitat requirements, with tiny seeds lacking endosperm, making natural germination difficult and its wild resources on the verge of extinction [22]. Non-symbiotic in vitro culture using a medium can promote rapid seed germination, and in basic medium, the appropriate use of plant growth regulators and exogenous additives can effectively promote the induction of explants. This study used MS as the basic medium and systematically explored the key stages from seed germination to rooting of in vitro seedlings of *D. flexicaule* by comparing and analyzing the effects of different concentrations of plant growth regulators and exogenous additives.

### 3.1. Analysis of the Effects of Different Concentrations of NAA and Potato Homogenate on the Germination of Seeds

When the fruits are ripe, morphological and anatomical analyses showed that the embryo is immature and undifferentiated in orchid seeds; the immature embryo needs cell division and differentiation by increasing its size [42,43]. Auxin governs processes such as seed dormancy and germination, cell division, bud differentiation, and root development by altering internal hormone concentrations and modulating associated enzymatic activity [44,45]. Auxins such as 2,4-dichlorophenoxyacetic acid (2,4-D), α-naph-thaleneacetic acid (NAA), and indole-3-butyric acid (IBA) are commonly used to induce callus formation and embryogenic competence [46,47]. Organic growth supplements also impact the in vitro regeneration, multiplication, and growth of orchid seedlings [22,32]. The results showed that in the primary culture, adding different concentrations of NAA and potato homogenate to the MS medium can promote the sterile germination of *D. flexicaule* seeds, seeds germinated to form protocorms, and the seed germination rate was relatively high in the MS medium supplemented with potato homogenate. The comparison of seed germination rates among all treatment groups showed that when the basic MS medium contains potato as an exogenous organic additive, the seed germination rate is generally high, reaching over 90%. Low concentration of NAA (0.2 mg/L) could promote the division and elongation of embryonic cells, and the natural nutrients such as Vitamin (B1 and B6), mineral elements (potassium, iron, and magnesium), carbohydrate and amino acids provided by potato homogenate could accelerate the germination process, which was consistent with the characteristic of seed germination in orchid plants relying on exogenous nutrients [22,48]. A high concentration of NAA (0.5 mg/L) might inhibit the differentiation of embryonic cells, leading to a decrease in the germination rate. When the concentration of NAA was 0.2 mg/L, and the concentration of potato homogenate was 80 g/L, the seed germination rate was the highest, reaching 98.33%. According to the current report, this finding was in line with prior studies reporting that application of mashed potato of suitable concentration of 100 g/L to the culture medium maximized the seed germination rate [22]. This medium formula was conducive to rapid seed germination and could efficiently induce the formation of protocorms. Further comparison of the germination time and growth status of seeds in several culture media revealed that the germination time of seeds in the culture medium containing potato homogenate was shorter. Moreover, as the concentration of exogenous additives increased, the growth rate of protocorms increased, and the individual became plump and dense.

### 3.2. Analysis of the Effects of Different Concentrations of 6-BA Combined with NAA on the Proliferation and Differentiation of Protocorms

The subculture stage is to promote the proliferation of the protocorms and their differentiation into clustered buds and seedlings. As plant hormones, cytokinins such as 6-benzylaminopurine (6-BA), zeatin (ZT), and kinetin (KT) play an important role in plant growth and development, including cell division and the formation and activity of shoot meristems in the subculture proliferation stages. Previous studies have reported that cytokinins play an essential role in plant morphogenesis by regulating root and shoot growth in combination with auxin; the common combination form is 6-BA (0.5–2.5 mg/L) and NAA (0.1–1.0 mg/L). The effects of different concentrations of NAA and 6-BA combinations on the proliferation and differentiation of the protocorms were significantly different [43,49]. The proliferation coefficient of cluster shoots of *D. moniliforme* was 6.1 times compared with the control group while MS medium supplemented with NAA (1.0 mg/L) and BA (1.0 mg/L) [43]. Xiong et al. [49] demonstrated that the proliferation coefficient of cluster shoots of *Flickingeria albopurpurea* was highest when MS medium supplemented with combinations of 6-BA (0.5 mg/L) and NAA (0.5 mg/L) was suitable for protocorm proliferation and differentiation. These results are consistent with findings in this study. Comprehensive analysis in this study indicated that the proliferation coefficient of protocorms in culture medium 4 (MS + 0.5 mg/L NAA + 0.5 mg/L 6-BA) was the largest, and the differentiation percentage (%) was relatively higher than those in most other media 2, 3, 5, and 6. Compared with previous studies [43,49], the results of this research indicated a modest proliferation coefficient, which might be related to the differences in exogenous organic additives. With the increase in the 6-BA concentration, when it exceeded 0.5 mg/L, the browning of the protocorms was aggravated, and the differentiation rate of the clustered buds decreased (for example, the differentiation rate of medium No. 6 was only 50%), indicating that high concentrations of cytokinin had an inhibitory effect on the proliferation of clustered buds [32,33,43]. Potato homogenate was demonstrated to effectively promote the proliferation and differentiation of protocorms, as it supplies high-quality proteins, starch, and essential minerals that support cell division and metabolism [50]. Nguyen et al. [51] found that raising the mashed potato concentration to 30 g/L resulted in higher shoot length and a higher number of leaves per plant in *D. nobile*. After adding potato homogenate to the basic medium 1/2MS, Liu et al. [22] found that protocorms formed by the germination of *D. flexicaule* seeds were large and robust, which was conducive to the formation of young plants, and indicated that potato homogenate also has a promoting effect on the differentiation of protocorms. Li et al. [52] proved that when 40–60 mL/L of potato homogenate is added to the MS medium for cultivation, the protocorms differentiated completely, and the differentiated seedlings were thicker and stronger, which was suitable for the differentiation of protocorms of *D.nobile* into seedlings. The results in the present study were consistent with the previous ones; although the proliferation coefficient of the blank control group CK was low when only 60 g/L of potato homogenate was added, the differentiation rate was high. It was speculated that in the absence of hormones, protocorms preferred organ differentiation rather than proliferation; exogenous additives could promote the differentiation of the protocorms. In addition, it was found that the buds differentiated from the protocorms had no yellowing phenomenon, indicating that the addition of 0.5 g/L activated carbon to the medium could reduce the degree of yellowing and senescence of the *D. flexicaule* seedlings.

### 3.3. Effects of Plant Growth Regulators on Vigorous Growth and In Vitro Rooting

Strengthening and rooting cultivation of tissue culture seedlings is a crucial step in the rapid propagation of orchids through in vitro culture. The evaluation of the growth potential of tissue culture seedlings mainly manifests as the number of roots and rooting percentage (%), root length, number of adventitious buds, number of leaves, and plant height. Exogenous auxin has a significant effect on root development by mediating the unidirectional canalization of endogenous auxin, which promotes the formation of adventitious roots and lateral roots, enhances root density and length [45]. In general, in vitro plant culture, auxins such as NAA and indole-3-butyric acid (IBA) are widely employed. Our research has also confirmed that when the concentration of 6-BA was lower, the number of roots per shoot and root length showed a significant increasing trend with the increasing concentration of NAA in culture media 1 to media 3. When the concentration of 6-BA increased from 0.5 mg/L to 1 mg/L, the rooting percentage, number of roots and root length of shoot clusters showed a significant downward trend, whereas the growth situation of the tissue culture seedlings such as number of leaves and average plant height per cluster showed more robust, which demonstrated that exogenous cytokinin as 6-BA has a negative regulatory function on root growth and antagonize adventitious rooting, stimulates bud proliferation and formation, and promotes stem growth [45]. Exogenous cytokinin promotes lateral root initiation and root hair formation by modulating hormone transport and signaling in pericycle and epidermal cells [53]; this was indicated by the number of roots and root length of medium 5.

Banana puree is frequently used as an organic additive in tissue cultures due to its high content of mineral elements, vitamins, and natural growth regulators (such as zeatin, gibberellin, and IAA), which are effective for promoting the growth of orchid seedlings in vitro propagation [22,32]. Activated carbon is commonly employed in in vitro culture systems for its capacity to adsorb excess PGRs and other inhibitory compounds, thereby promoting shoot elongation and physiological development [54]. The effect of additives on the growth and propagation efficiency of orchids has been researched; MS medium supplemented with 20 g/L mashed banana and 1 g/L activated charcoal was the most suitable medium for growth and development of in vitro *D. anosmum* [31]. In this study, during the vigorous growth and rooting stage of the tissue culture seedlings, 1/2MS medium was used while adding a consistent concentration of exogenous substances for banana puree and activated carbon. Comprehensive comparative analysis revealed that medium CK without growth regulators (6-BA and NAA) has a more prominent effect in promoting the growth of tissue culture seedlings. The results showed that, except for the average number of roots per tissue culture seedling, which was slightly lower than medium 5, all other indicators of CK medium used as the control group, such as average root length per plant, number of proliferating adventitious buds per plant, average number of leaves per plant, and average plant height, were significantly higher than other medium treatments. This indicated that tissue culture seedlings of *D. flexicaule* had a higher effect in rooting induction and more robust shoot growth performance cultured in 1/2MS culture medium without exogenous plant growth regulators (medium CK), but supplemented with 50 g/L banana puree and 1 g/L activated carbon as exogenous organic additives, which would be cheaper and lower the somaclonal-variation risk.

## 4. Materials and Methods

### 4.1. Test Materials

The capsule fruits of *D. flexicaule* were kindly provided by Laojie Ling *D. flexicaule* Ecological Plantation in November 2020, Xixia Ren’s *D. flexicaule* Agricultural Cooperative (Funiu Mountain, Nanyang, Henan Province, China; 111°44′5.37″ E, 33°35′39.67″ N, an altitude of 780 m). The *D. flexicaule* plants have been grown in an artificial wild environment for 3 years, where there is a river and trees within a 100-m radius around the plantation. The disease-free, fully mature golden-yellow capsules (fruit) were selected and collected. They are locally known as “golden melons”.

### 4.2. Disinfection of D. flexicaule Fruits

Pre-treatment of the fruits and acquisition of sterile seeds. Use a fine brush to gently remove the debris on the surface of the *D. flexicaule* fruits, then place them in a small beaker and rinse with running water for 1 h, soak in a diluted washing powder solution for 30 min, and rinse again with running water for 30 min. Transfer the washed fruits to the superclean bench for sterilization treatment. Previous studies showed that the pollution rate was relatively low and the germination rate was relatively high while fruits or seeds were surface sterilized with 75% ethanol and 0.1% HgCl_2_ [22,47]. Then sterilization treatment is as follows: First, immerse the materials in 75% alcohol solution for 30 s, take them out and rinse 5 times with sterile water; then immerse them in 0.1% mercuric chloride solution for disinfection for 10 min, and intermittently shake the solution with a glass cup during the disinfection process; then rinse 5 times or more with sterile water; finally, use sterile paper to absorb the residual moisture on the surface of the fruits and place them in an aseptic culture dish for use.

### 4.3. Inoculation of Explants and Induction of the Protocorms

Under sterile conditions, the sterilized fruits were cut longitudinally with a dissecting knife and picked up with forceps and gently shaken over the mouth of the wide-mouthed culture flask to evenly sprinkle the tiny, pale yellow seeds from the fruit shells onto the sterile culture medium, serving as explants for protocorm induction culture. The inoculation operation was completed in the area close to the alcohol lamp flame to reduce the infection of foreign bacteria. The protocorms induction culture medium was based on Murashige and Skoog (MS) medium [55], supplemented with 3% sucrose (Solarbio Science & Technology, Beijing, China), 0.7% agar (Sangon Biotech, Shanghai, China), 0.2 mg/L and 0.5 mg/L of auxin NAA (BIORIGIN, Beijing, China), and 40 g/L and 80 g/L of potato homogenate (Preparation method: select fresh, non-budded and disease-free potatoes, wash them thoroughly, peel and cut them into small pieces, weigh 40 g (80 g) and put them in a pot. Add an appropriate amount of distilled water, bring it to a boil, and then simmer over low heat until soft and tender. Filter out the residue with clean gauze and collect the filtrate. A blank control treatment CK (without hormones and potato homogenate) was also set up. All media containing auxin NAA were precisely adjusted to pH 6.0 using 1 mol/L NaOH or 1 mol/L HCl, and then followed by autoclaving at 121 °C (approx. 108 kPa) for 20 min. A total of five culture medium formulations were designed to detect the differences in the induction of protocorms by different culture medium conditions on *D. flexicaule* seeds (Table 1). Each treatment consisted of three independent biological replicates; five bottles were inoculated for each biological replicate.

All culture media in which the explants (seeds) have been inoculated were placed in a constant-temperature, lighted, sterile culture room for cultivation. The temperature is set at 25 ± 1 °C. In the early stage of cultivation, a sheet of shading paper is placed on the culture medium for continuous 5 days of dark cultivation, and then alternating light and dark cultivation is carried out under the conditions of 16 h light/8 h dark photoperiod provided by cool white fluorescent lighting with a photosynthetic photon flux density (PPFD) of 30 μmol m^−2^ s^−1^. After 3 weeks of cultivation, different fields of view are selected under a stereomicroscope to count the germination of 80 seeds for each biological replicate (seeds are counted as successfully germinated when they break through the seed coat). The germination rate of the *D. flexicaule* seeds is calculated. After 2 months of cultivation, seed germination and the induction of callus are observed, and data are statistically analyzed.

### 4.4. Multiplication and Differentiation of Protocorms

The protocorms induced in the initial seed germination culture were selected for multiplication and differentiation culture. Protocorms with vigorous growth, uniform shape, size, and color were selected from the initial seed germination induction culture as explants for inoculation. Under aseptic conditions, the clustered protocorms were separated into individual ones and inoculated onto sterile medium. During inoculation, the protocorms were placed with the protocorm tipping upward and evenly distributed on the medium, and gently pressed to ensure full contact with the medium. To investigate the effects of 6-BA (BIORIGIN, Beijing, China) in combination with NAA on multiplication and differentiation of protocorms, a two-factor experimental design was implemented using Murashige and Skoog (MS) medium [55]. The experiment comprised seven treatment combinations (Table 2). Formula treatments 1–3 added NAA (0.2 mg/L) and 6-BA (0.5 mg/L, 1.0 mg/L, 1.5 mg/L); formulas 4–6 added NAA (0.5 mg/L) and 6-BA (0.5 mg/L, 1.0 mg/L, 1.5 mg/L), and MS medium without growth regulators served as the control. All differentiation media were supplemented with 30 g/L sucrose, 60 g/L potato homogenate (The preparation method was the same as above), 0.5 g/L activated carbon (Solarbio Science & Technology, Beijing, China), and solidified with 0.7% (*w*/*v*) agar (Sangon Biotech, Shanghai, China). All media containing NAA and 6-BA were precisely adjusted to pH 6.0 using 1 mol/L NaOH or 1 mol/L HCl, and then autoclaved at 121 °C (approx. 108 kPa) for 20 min. Each treatment consisted of three independent biological replicates and 7 bottles of medium prepared for each biological replicate; each wide-mouthed culture flask was inoculated with 20 protocorms. The inoculated cultures were placed in an aseptic culture room for cultivation, with a constant temperature of 25 ± 1 °C and a 16 h light/8 h dark photoperiod provided by cool white fluorescent lighting with a photosynthetic photon flux density of 30 μmol m^−2^ s^−1^. After 60 days of culture, the multiplication (Proliferation Coefficient = Number of protocorms for proliferation/Number of protocorms for inoculation) and differentiation of the protocorms were observed and recorded.

### 4.5. Promotion of Vigorous Growth and Rooting Cultivation of Tissue Culture Seedlings

To evaluate the effects of growth regulator type and its concentration during rooting cultivation, two-factor experimental design was implemented using 1/2 MS [55] as the basal medium, which comprised seven treatment combinations involving 6-BA (0, 0.5 and 1.0 mg/L) and NAA (0, 0.2 and 0.5 mg/L), with the addition of 50 g/L banana puree (Preparation method: select fresh and ripe bananas, remove the banana skins; precisely weigh 50.0 g of the fruit flesh, add a small amount of distilled water, and use mortar to grind the fruit flesh into fine paste; Finally, use clean gauze to filter out coarse fibers), and 1 g/L activated carbon (Solarbio Science & Technology, Beijing, China). All media containing NAA and 6-BA were precisely adjusted to pH 6.0 using 1 mol/L NaOH or 1 mol/L HCl, and then followed by autoclaving at 121 °C (approx. 108 kPa) for 20 min. A system of 7 different combinations of plant growth regulators was designed for the promotion of vigorous growth and rooting induction of *D. flexicaule* tissue culture seedlings, and MS medium without growth regulators served as the control (Table 4). Selected sterile seedlings without roots and with a uniform height of about 1.5 ± 0.2 cm and 2 complete true leaves from the subculture were used, and the morphological lower end of the explant was inserted into the culture medium (0.5 ± 0.1) cm deep at a vertical angle. Each treatment consisted of three independent biological replicates and 6 bottles of medium prepared for each biological replicate; each culture flask was uniformly inoculated with 6 healthy seedlings. All experiments were performed under sterile conditions to prevent contamination. The inoculated *D. flexicaule* tissue culture seedling materials were placed in the sterile culture room and cultured under a constant temperature of 25 °C. First, they are dark-cultured (covered with shading paper) for one week, and then cultivated for 60 days under a 16 h light/8 h dark photoperiod provided by cool white fluorescent lighting with a photosynthetic photon flux density of 30 μmol m^−2^ s^−1^. Observe and record the growth conditions, statistically calculate the number of roots and rooting percentage (%), root length, number of adventitious buds, number of leaves, and plant height.

### 4.6. Statistical Analysis

Using Excel 2019 to conduct data statistics for the following parameters, the number and germination rate of the seeds, proliferation coefficient and differentiation rate of the protocorm, and the effects of different ratios of plant growth regulators on the rooting induction culture of tissue culture seedlings (rooting rate, the number of leaves per group culture seedling, the number of roots, average root length, the number of proliferating buds, and plant height). Each variable was separately analyzed; data were analyzed using one-way and two-way analysis of variance (ANOVA) with a post hoc Duncan’s Multiple Range Test (DMRT), with the results considered statistically significant at *p* ≤ 0.05. Percentage data were subjected to arcsine square-root transformation prior to analysis to meet the assumptions of normality and homogeneity of variance. Statistical analyses of variance were performed using IBM SPSS Statistics version 22.0 (IBM Corp., Armonk, NY, USA). Significant differences are indicated by different lowercase letters.

## 5. Conclusions

The plants of *Dendrobium* have small seeds. When their fruits mature, the seed embryos are not fully developed and lack endosperm, resulting in an extremely low germination rate under natural conditions. This study successfully conducted programmed experiments as follows: sterile germination of *D. flexicaule* seeds, the proliferation and differentiation of protocorms, and the growth and rooting of regenerated seedlings, expecting to establish an efficient plant regeneration system of *D. flexicaule*. Based on the seeds of the endangered medicinal plant *D. flexicaule*, the results of this study identified the key regulatory factors for the growth and development at each stage of tissue culture of *D. flexicaule*, which provided effective and relatively successful reference for the establishment of an in vitro rapid propagation system of *D. flexicaule*. Despite these advancements, further refinement of the regeneration system is warranted, especially regarding the verification data for the ex vitro acclimation and transplantation of tissue culture seedlings. The tissue culture seedlings of *D. flexicaule* obtained through seed germination and in vitro tissue culture would be important supplementary and genetic resources for wild endangered *Dendrobium* species. Genetic variation analysis can be conducted among protocorm-derived lines to estimate clonal uniformity and obtain stable active substances in the medicinal plant. This study establishes an effective protocol for in vitro tissue culture of *D. flexicaule* and offers valuable technical support for the establishment of a rapid regeneration propagation system in future research, which is expected to protect wild *Dendrobium* resources and achieve sustainable utilization of the medicinal plant resources.

## Figures and Tables

**Table 1:** Effects of different concentrations of hormones and exogenous additives on the germination of *D. flexicaule* seeds.

No.	Additives		Germination Percentage	Appearance of Seed Germination	Morphology of Protocorms
	NAA (mg/L)	Potato Homogenate (g/L)			
CK	0	0	87.50 ± 5.45 ^c^	Germinate in 25 days and grow slowly.	Most of them are yellow callus tissues, only a few are conical protocorms.
1	0.2	40	95.83 ± 2.60 ^ab^	Germinate in 18 days and grew normally.	Green protocorms, most of which are plump.
2	0.2	80	98.33 ± 1.44 ^a^	Germinate in 15 days and grew vigorously.	Green protocorms, most of which are large, plump, and conical.
3	0.5	40	90.83 ± 1.44 ^bc^	Germinate in 20 days and grow slowly.	Yellow–green protocorms are plump and round, with a few being larger and conical.
4	0.5	80	93.33 ± 1.91 ^ab^	Germinate in 18 days and grew normally.	Light green protocorms are plump and round, with a few being conical.

Note: Data in the table are presented as mean ± standard deviation (SD). The data marked with different lowercase letters within the same column indicate significant differences between treatments (*p* < 0.05).

**Table 2:** Effects of different concentrations of 6-BA combined with NAA on the proliferation and differentiation of protocorms.

Medium No.	Exogenous Plant Hormones		Proliferation Coefficient	Differentiation Percentage (%)	Appearance of Proliferation and Differentiation of Protocorms
	NAA (mg/L)	6-BA (mg/L)			
CK	0	0	0.77 ± 0.11 ^c^	100 ± 0.00 ^a^	Dark green protocorms proliferate sparingly, and all differentiate into robust seedlings, which induce numerous adventitious roots.
1	0.2	0.5	0.90 ± 0.08 ^bc^	86.33 ± 1.15 ^ab^	Green protocorms have few divisions and little browning, and most of them differentiated into seedlings.
2	0.2	1.0	1.67 ± 0.68 ^a^	74.33 ± 11.85 ^b^	Green protocorms have dense proliferation and less browning; some of them differentiated into vigorous seedlings.
3	0.2	1.5	1.50 ± 0.34 ^ab^	73.33 ± 13.50 ^b^	Green protocorms have dense proliferation and less browning; some of them differentiated into ordinary seedlings.
4	0.5	0.5	1.88 ± 0.31 ^a^	81.33 ± 2.89 ^b^	Dark green protocorms have dense proliferation and less browning; most of them differentiated into stronger seedlings.
5	0.5	1.0	1.33 ± 0.28 ^abc^	57.67 ± 8.39 ^c^	Pale green protocorms have dense proliferation and obvious browning; a few differentiated into seedlings.
6	0.5	1.5	1.52 ± 0.19 ^ab^	50.00 ± 8.66 ^c^	Pale green protocorms have dense proliferation and severe browning; a few differentiated into seedlings.
two-way ANOVA	*F* NAA		*p* = 0.194	*p* = 0.002 **	
	*F* 6-BA		*p* = 0.795	*p* = 0.001 ***	
	*F* NAA × 6-BA		*p* = 0.014 **	*p* = 0.148	

Note: Data in the table are presented as mean ± standard deviation (SD). The data marked with different lowercase letters within the same column indicate significant differences between treatments (*p* < 0.05). ** indicates significant differences at *p* < 0.05, *** indicates extremely significant at *p* ≤ 0.001 level.

**Table 3:** Effects of different concentrations of plant growth regulators on vigorous growth and in vitro rooting of *D. flexicaule* tissue culture seedlings.

Culture Medium No.		Rooting Percentage (%)	Number of Roots	Root Length (cm)	Number of Adventitious Buds	Number of Leaves	Plant Height (cm)	Appearance of Tissue Culture Seedlings
CK		100%	5.09 ± 0.38 ^b^	2.03 ± 0.15 ^a^	1.70 ± 0.77 ^a^	12.24 ± 0.37 ^b^	2.37 ± 0.39 ^a^	Many long roots, the plants are robust.
1		100%	3.44 ± 0.30 ^c^	1.09 ± 0.04 ^de^	1.02 ± 0.27 ^ab^	8.83 ± 1.26 ^d^	2.36 ± 0.24 ^a^	Many roots and the plants are robust.
2		100%	4.61 ± 0.39 ^b^	1.33 ± 0.09 ^cd^	1.54 ± 0.09 ^a^	10.35 ± 0.72 ^c^	2.22 ± 0.18 ^a^	Many roots and the plants are relatively short.
3		100%	4.76 ± 0.69 ^b^	1.46 ± 0.25 ^c^	0.52 ± 0.94 ^b^	14.26 ± 0.99 ^a^	2.42 ± 0.03 ^a^	Many roots, numerous leaves, and the plants are relatively robust.
4		94.3%	1.35 ± 0.27 ^e^	0.67 ± 0.05 ^f^	0.82 ± 0.26 ^ab^	13.26 ± 0.96 ^ab^	2.45 ± 0.23 ^a^	A few roots and the roots are relatively short.
5		100%	5.98 ± 0.23 ^a^	1.75 ± 0.20 ^b^	1.00 ± 0.39 ^ab^	10.67 ± 0.45 ^c^	2.14 ± 0.23 ^a^	The number of roots is the greatest, and the plants are robust.
6		98.1%	2.30 ± 0.28 ^d^	0.87 ± 0.08 ^ef^	1.19 ± 0.12 ^ab^	12.39 ± 0.73 ^b^	2.20 ± 0.34 ^a^	A few roots, numerous leaves, and the plants are robust.
	*F* NAA		*p* = 0.000 ***	*p* = 0.000 ***	*p* = 0.297	*p* = 0.004 **	*p* = 0.346	
two-way ANOVA	*F* 6-BA		*p* = 0.000 ***	*p* = 0.000 ***	*p* = 0.028 **	*p* = 0.015 **	*p* = 0.844	
	*F* NAA × 6-BA		*p* = 0.000 ***	*p* = 0.000 ***	*p* = 0.192	*p* = 0.001 ***	*p* = 0.618	

Note: Data in the table are presented as mean ± standard deviation (SD). The data marked with different lowercase letters within the same column indicate significant differences between treatments (*p* < 0.05). ** indicates significant differences at *p* < 0.05, *** indicates extremely significant at *p* ≤ 0.001 level.

**Table 4:** Rooting induction medium for tissue culture seedlings of *Dendrobium flexicaule* supplemented with different ratios of plant growth regulators.

Culture Medium No.	Basic Culture Medium	Agar (g/L)	Sucrose (g/L)	Banana Puree (g/L)	Activated Carbon (g/L)	6-BA (mg/L)	NAA (mg/L)
CK	1/2MS	7	30	50	1	0	0
1	1/2MS	7	30	50	1	0.5	0
2	1/2MS	7	30	50	1	0.5	0.2
3	1/2MS	7	30	50	1	0.5	0.5
4	1/2MS	7	30	50	1	1	0
5	1/2MS	7	30	50	1	1	0.2
6	1/2MS	7	30	50	1	1	0.5

Note: MS: Murashige and Skoog; 6-BA: 6-Benzylaminopurine; NAA: 1-naphthylacetic acid.

## Data Availability

The original contributions presented in this study are included in the article. Further inquiries can be directed to the corresponding author.
